# Reply to the comment on “Trends in rates of orthopedic surgery in Germany: the good, the bad, the ugly.”

**DOI:** 10.1007/s10198-019-01154-y

**Published:** 2020-01-21

**Authors:** Natalie Baier, Lisa-Marie Sax, Leonie Sundmacher

**Affiliations:** 1 Department of Health Care Management, Berlin Centre for Health Economics Research (BerlinHECOR), Technische Universität Berlin, Str. des 17. Juni 135, 10623 Berlin, Germany; 2 City University London, Northampton Square, Clerkenwell, London, EC1V 0HB UK; 3 Department of Health Services Management, Ludwig-Maximilians-Universität München, Schackstraße 4, 80539 Munich, Germany

We wish to thank the Editor for giving us the opportunity to respond to the points concerning the statistical specification and the analytical approach raised by Prof. Afschin Gandjour.

Gandjour criticizes that the dependent variable is not defined. However, we described the dependent variable, the number of hip replacements, knee replacements and spine surgeries, comprehensively in the methodology section in the subsection “data” on page 164. It is furthermore pointed out that the dependent variable Y appears on both sides of the Eqs. 3 and 4 on page 168. This is true and it is an approach to model spatial dependence (see analysis section on page 167). The employed spatial Durbin model includes a spatially lagged variable of the dependent variable. The dependent variable for one district is in this case affected by the dependent variable for the other districts. We included the lagged variable as we assumed potential spillover effects caused by, for example, communication between physicians of neighboring districts. Detailed information on the modeling of spatial dependencies is given in Ward and Gleditsch [1] and Elhorst [2]. In this context, the comment that we used an OLS fixed effects model is not correct. Our regression model is a spatial panel model combining the Mundlak Model and the spatial Durbin model. The equation is presented on page 168. This type of model allows to control for individual heterogeneity and spatial dependence.

Furthermore, the concern is raised that we might have a problem with reverse causality, as “an increased volume of surgical procedures in some hospitals may encourage other providers to enter the market and offer these procedures as well, thus causing more competition”. This refers to our hypothesis that increased competition, measured among others by the number of hospitals, is associated with increased procedure volume. The number of hospitals is determined by hospital planning which is done by the ministries of health at the federal state level. Methods for planning differ between the federal states. Regulation of hospital capacities is planned on the basis of principles of need and performance [3]. A closer look at the federal state of Berlin shows that the hospital plan of 2010 was the binding basis until 2015. The hospital plan for 2016 was developed in a 3-year planning process with the participation of relevant actors in the hospital sector [4]. Only hospitals included in the hospital plan are reimbursed by the sickness funds and receive capital investments by the federal states [3]. Due to this regulation, it is unlikely that other providers enter the market to offer procedures for which we observe an increasing volume.

Finally, it is mentioned that the introduction and the analysis are disconnected from the conclusion. According to Gandjour’s understanding of our article, the analysis was supposed to be about unnecessary surgeries (indication quality) and in the concluding section, specialization of hospitals are discussed (procedural quality). We think that there might be a misunderstanding regarding the aim of the analysis. Based on the theory and empirical studies presented in the introduction of our article, we hypothesized that the age- and sex-standardized rates of surgical procedures are positively associated with the degree of competition for these procedures. Admitting patients for potentially unnecessary services is listed as a potential strategy for hospitals to increase the number of patients. We had to reject our hypothesis as we found that with increasing market concentration, the rates of hip and knee replacement rose. Specialization is discussed as a potential reason for this result. The data set for the analysis was chosen to analyze trends and regional variation on a nationwide level. Our aim was not to analyze the individual quality of the procedures (indication or procedural).

## References

[1] Ward MD, Gleditsch KS (2019). Spatial Regression Models.

[2] Elhorst JP (2010). Applied spatial econometrics: raising the bar. Spat. Econ. Anal..

[3] Busse, R., Blümel, M.: Germany: health system review. Health System in Transition. 2014 25115137

[4] Senatsverwaltung für Gesundheit, Pflege und Gleichstellung. Krankenhausplan des Landes Berlin. (2016). https://www.berlin.de/sen/gesundheit/themen/stationaere-versorgung/krankenhausplan/. Accessed 12 Oct 2019

